# Development of orthotopic tumour models using ultrasound-guided intrahepatic injection

**DOI:** 10.1038/s41598-019-46410-6

**Published:** 2019-07-09

**Authors:** L. E. McVeigh, I. Wijetunga, N. Ingram, G. Marston, R. Prasad, A. F. Markham, P. L. Coletta

**Affiliations:** 1 Leeds Institute of Medical Research, St James’s University Hospital, Leeds, LS9 7TF UK; 2 Department of Hepatobiliary and Transplant Surgery, St. James’s University Hospital, Leeds, LS9 7TF UK

**Keywords:** Colorectal cancer, Bile duct cancer, Experimental models of disease, Cancer imaging

## Abstract

Mouse models of human diseases are an essential part of the translational pipeline. Orthotopic tumour mouse models are increasingly being used in cancer research due to their increased clinical relevance over subcutaneous xenograft models, particularly in relation to metastatic disease. In this study, we have developed orthotopic colorectal cancer liver metastases (CRCLM) and primary cholangiocarcinoma (CCA) models in BALB/c nude mice using minimally invasive ultrasound-guided intrahepatic injection. Due to its minimally invasive nature, the method reduced risk from surgical complications whilst being fast and easy to perform and resulted in measurable tumour volumes 1 to 3 weeks post-injection. Tumour volumes were monitored *in vivo* by weekly high-frequency ultrasound (HF-US) and/or twice weekly bioluminescence imaging (BLI) and confirmed with end-point histology. Take rates were high for human CRC cells (>73%) and for CCA cells (90%). We have demonstrated that this method reliably induces CRCLM and CCAs, in which tumour volume can be monitored throughout using HF-US and/or BLI. This provides a promising experimental tool for future testing of cancer therapeutics in an orthotopic model.

## Introduction

Metastatic cancer is the leading cause of death for patients diagnosed with colorectal cancer (CRC) with a five-year survival of stage-IV disease at diagnosis just 7–8%^1^. The most common site of metastasis is the liver, amounting to 70% of all cases^2^. Cholangiocarcinoma (CCA) although relatively rare, has a five-year survival of approximately 5%^3^. Both are characterised as being difficult to treat with poor long-term survival of patients. Therefore, there is a pressing need for more reliable models of these diseases for pre-clinical evaluation of anti-cancer therapies. Currently, orthotopic models recapitulating these human cancers are time-consuming to establish with poor or unreliable tumour formation.

In CRC, cancer cells from the primary tumour disseminate via the haematogenous route to secondary sites in the liver^4,5^. Mouse models of CRC and/or liver metastases are either transplantable or genetically modified, with the latter generally taking a long time to establish and rarely metastasise^6,7^. Transplantable models are much faster to generate but involve direct surgical implantation of cancer cells or tissues into the caecum or spleen, or direct injection into the portal vein^8–11^. The most commonly exploited models of colorectal cancer liver metastases (CRCLM) are injection site-dependant, either by surgical inoculation of the spleen or caecum involving implantation or subserosal injection^12,13^. Intrasplenic injection results in the rapid formation of liver tumours by drainage into the hepatic portal vein via the splenic vein but often involves the removal of the spleen; a vital immune organ. Caecal injections are technically more challenging with a higher risk of post-surgical complications, but with the advantage of allowing the study of invasion from the primary site. However, this results in the slow (up to four months) and unreliable formation of metastases in the liver in addition to lung, lymph nodes and peritoneal tumours^10^.

Pre-clinical orthotopic models of CCA generally involve chemotoxic agents or gene knock-out techniques^14,15^. The process of generating these models is generally long and expensive. A syngeneic rat model of intrahepatic CCA using a rat-derived CCA cell line surgically implanted into the left hepatic duct with/without surgical ligation of the bile duct, reported 100% success rates^16^. Although this model has the advantage of producing bile duct obstruction (which is a common feature of CCA), the development of the model involved invasive surgery and does not utilise human-derived cancer cells.

The tumour microenvironment is thought to play an important role in influencing the growth, response to treatment and tumour immune responses *in vivo*^17–19^. Tumours grown subcutaneously do not accurately represent the natural tumour microenvironment which contains fibroblasts, immune cells and an extracellular matrix which have all been shown to be influencing factors for tumour establishment and growth^20^. Subcutaneous models, in general, have been poor predictors of drug efficacy in humans and therefore the need for more predictive models of human cancers^21^. Hence, the generation of *in vivo* models of advanced disease in an orthotopic setting are necessary for more effective translation of novel therapies in order to improve treatment outcomes. Immune compromised or competent mice can be used depending on the cell lines used (patient-derived and/or human xenografts or syngeneic grafts) for testing of cytotoxic or immunotherapies, respectively.

To this end, we have developed a minimally-invasive method to create orthotopic liver tumours that recapitulate cancers in the liver. Ultrasound-guided percutaneous injections were used to develop a pre-clinical orthotopic model of CRCLM, which was minimally-invasive, fast and easy to perform and was amenable to monitoring tumour growth regularly and economically via HF-US. The human colorectal adenocarcinoma cell line, SW620, that was first derived from a lymph node metastasis was used with or without Matrigel in BALB/c nude mice to assess the feasibility of generating CRCLM. TFK-1, an extra-hepatic CCA cell line transfected to express luciferase (TFKLuc2B2), was used for the generation of an orthotopic CCA model which had the advantage of longitudinal tumour growth monitoring using BLI as well as HF-US imaging.

## Results

### Development of an orthotopic model of CRCLM

**Figure 1:**
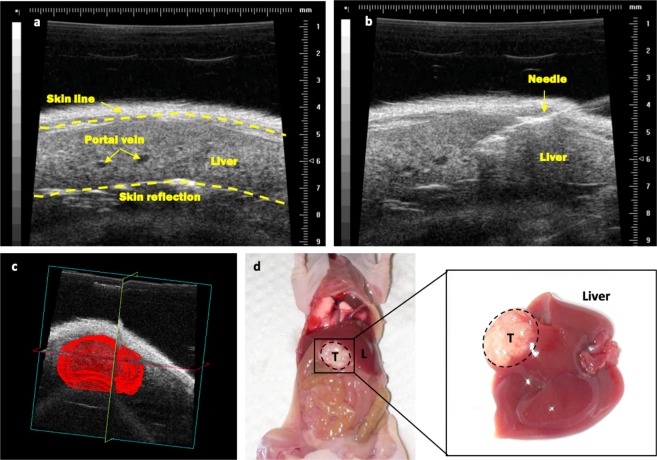
Representative images of ultrasound-guided intrahepatic injections and subsequent tumour. (**a**) HF-US image of mouse liver pre-injection. Skin reflection is an artefact indicated by the dashed line. (**b**) Intrahepatic injection of a 40 µl bolus of human CRC cells using a 29-gauge needle to a depth of 3 mm. Care was taken to avoid major blood vessels. (**c**) A 3D reconstruction (matrix) of the final orthotopic tumour volume 35 days after tumour inoculation. The 3D scans of tumours were performed by HF-US using a 40 MHz (RM-704) transducer on a motorised arm, with the VisualSonics Vevo 770 system. The 3D reconstructions were generated post-acquisition using the Vevo 770 version 3 software. (**d**) Dissection of the same mouse revealed no peritoneal disease, with normal organ colour and an obvious single tumour at injection site. Enlarged region shows the dissected liver with an orthotopic tumour. T = tumour, L = liver (tumour region circled with dashed line).

An orthotopic human CRCLM mouse model was developed using ten 5-week-old female BALB/c nude mice. Mice were randomised into two groups of five and anaesthetised with 5% (v/v) isoflurane gas in medical air at a flow rate of 2 L/min via an induction chamber, then positioned supine on the ultrasound platform with a nose cone for and maintained at 3% (v/v). Ultrasound gel was applied liberally to the abdomen and mice were imaged in B mode (2D) using a 40 MHz (RM-704) transducer with the VisualSonics Vevo 770 system, to determine and visualise the largest liver lobe (Fig. 1a). A 1 ml Terumo Myjector 29-gauge insulin syringe with a fixed 1.27 cm needle (Terumo, Tokyo, Japan) was aligned and advanced into the liver by freehand using the ultrasound monitor for reference. A 40 µl bolus of 20,000 SW620 CRC cells suspended in either PBS:Matrigel (1:1 v/v) or PBS alone was injected directly into the liver at a depth of 3 mm (Fig. 1b). A deliberate 30-second pause before the needle was retracted allowed for complete delivery of cells into the liver, to prevent recoil of cells and leakage into the peritoneum. A video showing an ultrasound-guided injection is included in the Online Resource 1.

Post-injection, anaesthesia was ceased and mice were placed in a warm recovery chamber and observed until fully recovered then returned to their original housing. The whole procedure from induction of anaesthesia to recovery took less than five minutes. Animals’ heart and respiration rates were monitored during the entire procedure through electrode pads on the ultrasound platform using the Vevo’s Physiological Controller Unit. Post-operative analgesics were not necessary with such a minimally-invasive technique. One mouse from the Matrigel group did not recover post-procedure for unknown reasons.

Due to the internal location of the tumours, tumour volume was monitored by HF-US imaging. The condition of each animal was monitored by daily visual inspection for clinical signs of pain or distress such as abnormal behaviours, piloerection, reduced mobility, body posture or isolation from cage mates. The Mouse Grimace Scale was also used to assess each animal for signs of pain. All indicators suggested that the animals were in minimal discomfort, if any, after this technique. Animals were weighed weekly and if they lost >10% of pre-experimental body mass, would be monitored daily. If weight loss reached 20%, animals would be humanely euthanised, however, in this case, all animals remained at a healthy weight throughout.

### Orthotopic CRCLM tumour growth

**Figure 2:**
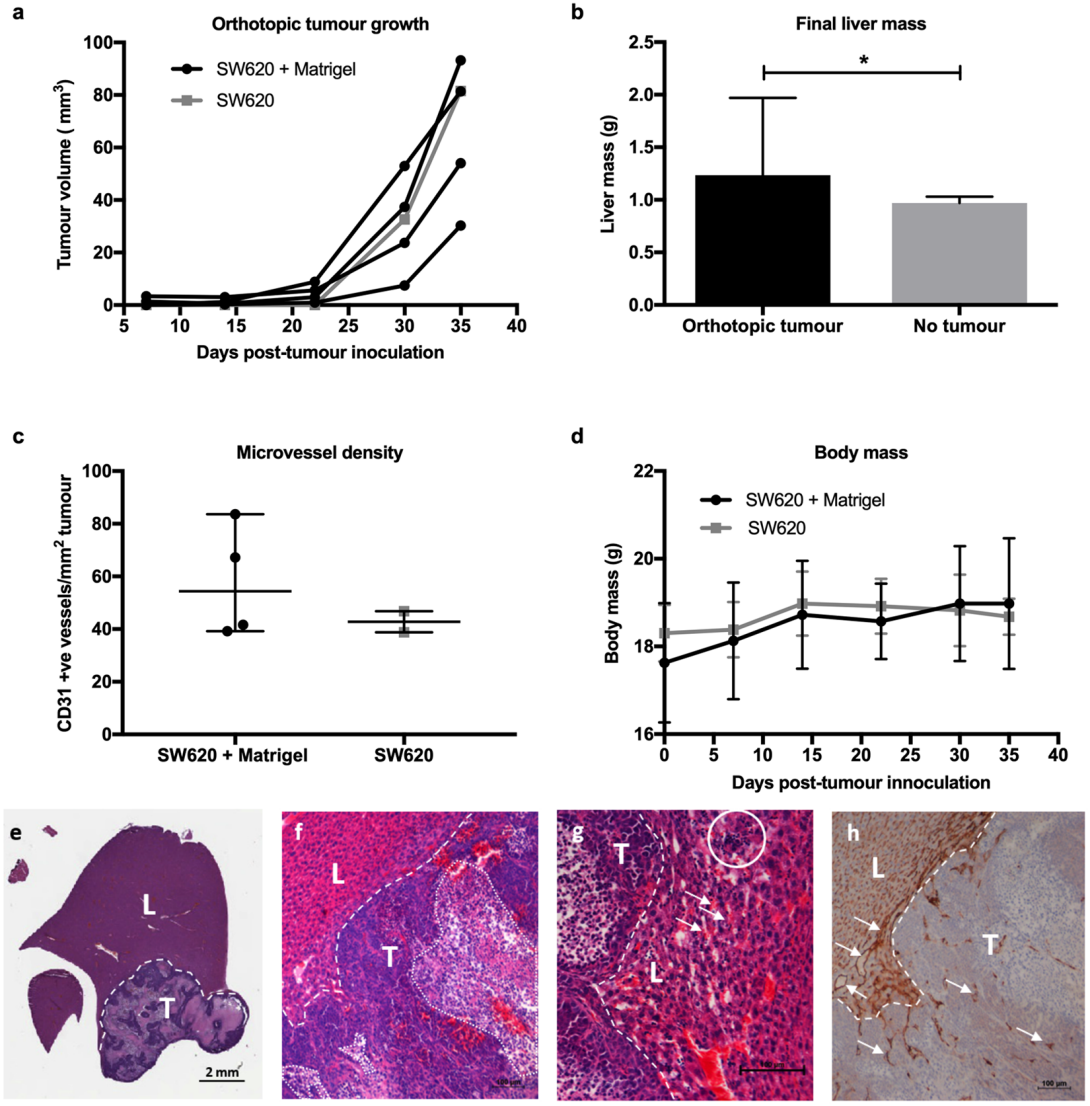
*In vivo* and *ex vivo* analysis of orthotopic SW620 liver tumour growth. (**a**) Graph of orthotopic growth measured *in vivo* by HF-US. (**b**) End-point liver mass (median ± 95% confidence interval) was statistically significantly different from livers with orthotopic tumours compared to those with no tumours *p = 0.048 (Mann-Whitney two-tailed). (**c**) Microvessel density was determined using the endothelial cell marker CD31 to immunolabel tumour blood vessels (median ± 95% confidence interval). (**d**) The body mass of the mice throughout the experiment (mean ± standard deviation). (**e**–**g**) Mouse livers with SW620 human CRC tumours were stained with H&E to reveal invasive boarders (dashed line) between liver (L) and tumour (T). Necrotic regions of tumour were common using this cell line (dotted line). (**g**) Red blood cells pooling in sinusoids (arrows) with inflammatory infiltrate (circled). (**h**) The endothelial cell marker CD31 was used to immunolabel blood vessels (arrows) of the tumour and liver and showed that the liver provided a highly vascularised microenvironment for the CRCLM tumour.

Orthotopic tumours were imaged weekly by HF-US for longitudinal monitoring of tumour growth. 3D-mode imaging acquisition was used, with the minimum step size possible for the length of tumour. The 3D reconstructions were generated post-acquisition using the Vevo 770 version 3 software (Fujifilm VisualSonics). Tumour volume was calculated by outlining the tumour area of multiple 2D sections at regular intervals by-hand throughout the image scan, which generated a 3D reconstruction and tumour volume. Animals were euthanised 35 days post-inoculation. Figure 1c shows an HF-US image of mouse liver bearing a single tumour acquired at end-point. After 35 days, mice had large singular tumours (Fig. 1d) with obvious distended abdomens. Tumour volumes were plotted over time (Fig. 2a) and final volumes are shown in Table 1. Based on *in vivo* HF-US imaging, the orthotopic xenograft take rate was 100% using Matrigel (after exclusion of the procedure-related mortality) and 20% without. One of the tumours without the use of Matrigel grew lower down in the liver and was out of the imaging region and upon dissection take rate was recalculated to 40%. Tumour doubling times for tumours with or without Matrigel were 3.9 ± 1.2 days compared to 3.4, respectively, with no statistically significant differences between groups, suggesting Matrigel did not influence tumour growth rate in this model. End-point liver mass was used to determine any significant differences between livers with orthotopic tumours and those with no tumours (Fig. 2b). Although small numbers of animals were used in this study, the significant difference obtained suggests the robustness of this technique. Livers with tumours had a significantly higher mass than those without, p = 0.048 (Mann Whitney, two-tailed). Tumour microvessel density was determined and was not statistically significantly different between groups, suggesting Matrigel did not influence blood vessel formation after 35 days (Fig. 2c). Body mass prior to procedure up to end-point was not statistically significantly different at any point during the experiment, indicating good health and well-being throughout (Fig. 2d).

**Table 1:** 3D volume measurements of orthotopic CRC tumours by HF-US, take rates and tumour doubling time. Take rates and final tumour volumes 35 days after tumour inoculation. N/R = non-recovery from anaesthesia, N/O = tumour not observed by US due to low position, N/T = no tumours. Median value given where possible.

Mouse	Tumour volume (mm^3^)	
	With Matrigel	Without Matrigel
1	93.2	81.6
2	81.4	N/O
3	54.1	N/T
4	30.3	N/T
5	N/R	N/T
Take rate	4/4 (100%)	2/5 (40%)
Median volume (mm^3^)	67.7	81.56
Median doubling time (days)	3.9	3.4

### Histological examination of CRCLM

By histology, tumours were observed in 4/4 (100%) of livers injected with cells in Matrigel and 2/5 (40%) without Matrigel (Table 1). The use of Matrigel may initially hold cells at the injection site by the formation of a plug and prevent leakage as seen where HF-US imaging missed one tumour due to its low positioning, potentially due to this reason.

Figure 2e–g show representative H&E staining of mouse livers containing an orthotopic tumour. Livers were analysed by H&E staining of paraffin-embedded fixed tissue and revealed single tumours with distinct liver-tumour borders. Orthotopic tumours characteristically had necrotic islands and areas of geographic necrosis with poor glandular differentiation and mainly formed sheets of epithelial cells, typical of SW620 tumours^22^. Invasive borders into the liver parenchyma were common, with inflammatory infiltrate and blood pooling in the sinusoids of the liver an indication of potential vascular congestion, which is often seen with liver cancers^23^ (Fig. 2g). The endothelial cell marker CD31 was used to immunolabel blood vessels of the tumour and liver and indicated how much more vascularised liver was compared to tumour (Fig. 2h).

### Independent validation of the CRCLM model

**Figure 3:**
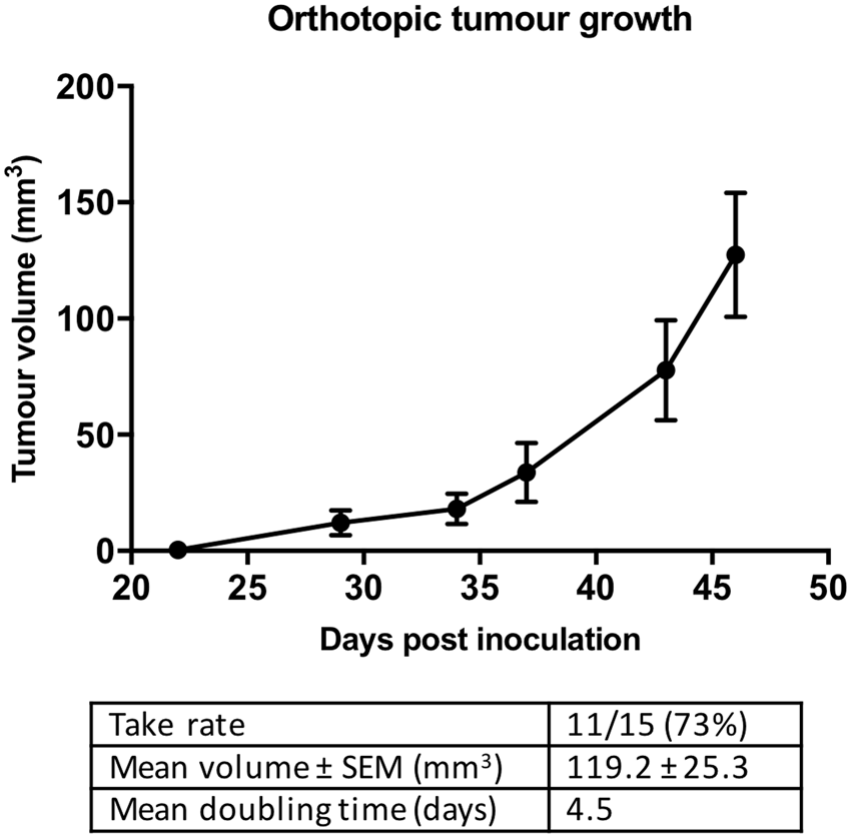
Orthotopic CRCLM tumour growth curve. This technique was independently validated by a second ultrasound operator using Matrigel with ultrasound-guided intrahepatic injection of SW620s and resulted in a take rate of 73% (11/15). Weekly HF-US imaging was used for longitudinal monitoring of tumour growth. At 47 days post-inoculation (end-point), tumour volume was determined as 119.2 ± 25.3 mm^3^ (mean ± SEM) with a mean tumour doubling time of 4.5 days.

This ultrasound-guided injection technique was independently validated by a second ultrasound operator using 15 5 to 7-week-old male BALB/c nude mice using Matrigel with the injection. All mice survived inoculation with weight monitoring and general health showing the procedure was well tolerated throughout. Animals were euthanised 47 days post-inoculation. This second cohort resulted in a take rate of 73% (11/15). Weekly HF-US imaging was used for longitudinal monitoring of tumour growth (Fig. 3). Mean (± SEM) tumour volume of 119.2 (± 25.3) mm^3^ was determined at end-point, with a mean tumour doubling time of 4.5 days.

### Development of an orthotopic model of primary cholangiocarcinoma (CCA)

**Figure 4:**
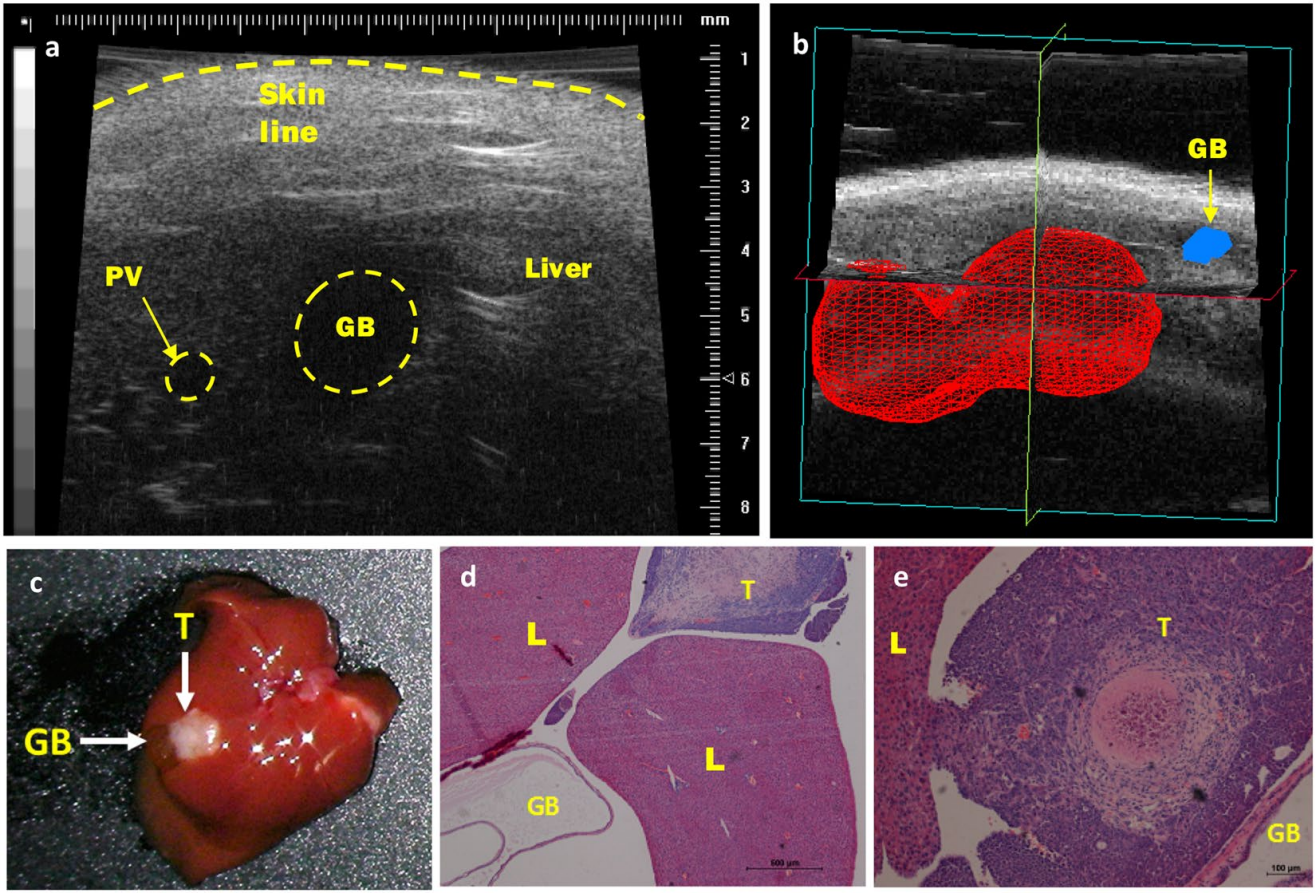
Representative images of HF-US imaging and subsequent orthotopic CCA tumours. (**a**) HF-US image of mouse liver prior to inoculation. The gallbladder (GB) was used as a landmark for injection. Portal vein (PV) also shown. (**b**) 3D reconstruction (matrix) of orthotopic tumour volume (day 14 post-inoculation) with gallbladder (arrow). 3D scans of tumours were performed using HF-US using a 40 MHz (RM-704) transducer on a motorised arm, with the VisualSonics Vevo 770 system. The 3D reconstructions were generated post-acquisition using the Vevo 770 version 3 software. (**c**) Representative mouse liver at end-point (19 days). Tumour (T) in close proximity to gallbladder (GB) indicated. (**d**,**e**) Liver (L) sections of two orthotopic tumours stained with H&E to demonstrate proximity of orthotopic tumour (<2 mm) to gallbladder (GB).

With the success and ease of this method for generation of CRCLM, the same technique was implemented to produce an orthotopic model of primary CCA. Ten 6 to 10-week-old female BALB/c nude mice were injected as described above with a 50 µl bolus of 5 million TFKLuc2B2 cells in PBS using HF-US (Matrigel not used). The gallbladder was used as a landmark for ultrasound-guided percutaneous injection into the liver as it was the closest relevant structure to the bile duct which could be located using ultrasound (Fig. 4a and Online resource 2). Animals were euthanised 19 days post-inoculation, and at end-point tumour take rate was 90% (9/10), two animals also had discrete intra-peritoneal tumours and one of these also had a subcutaneous tumour at the injection site (Online Resource 3). HF-US imaging was performed weekly and the median (± range) tumour volume was determined as 21.2 (9.0–32.5) mm^3^ 14 days post-injection (n = 6). Figure 4b shows a representative HF-US 3D tumour reconstruction image with the gallbladder nearby. At end-point, mice had tumours in the liver in close proximity to the gallbladder (Fig. 4c) and histological evaluation confirmed the presence of intrahepatic tumours close (<2 mm) to the gallbladder (Fig. 4d,e). One mouse was sacrificed at day 16 due to weight loss and abnormal behaviour. An orthotopic tumour was evident at necropsy with no obvious metastatic burden or cause of sudden deterioration.

**Figure 5:**
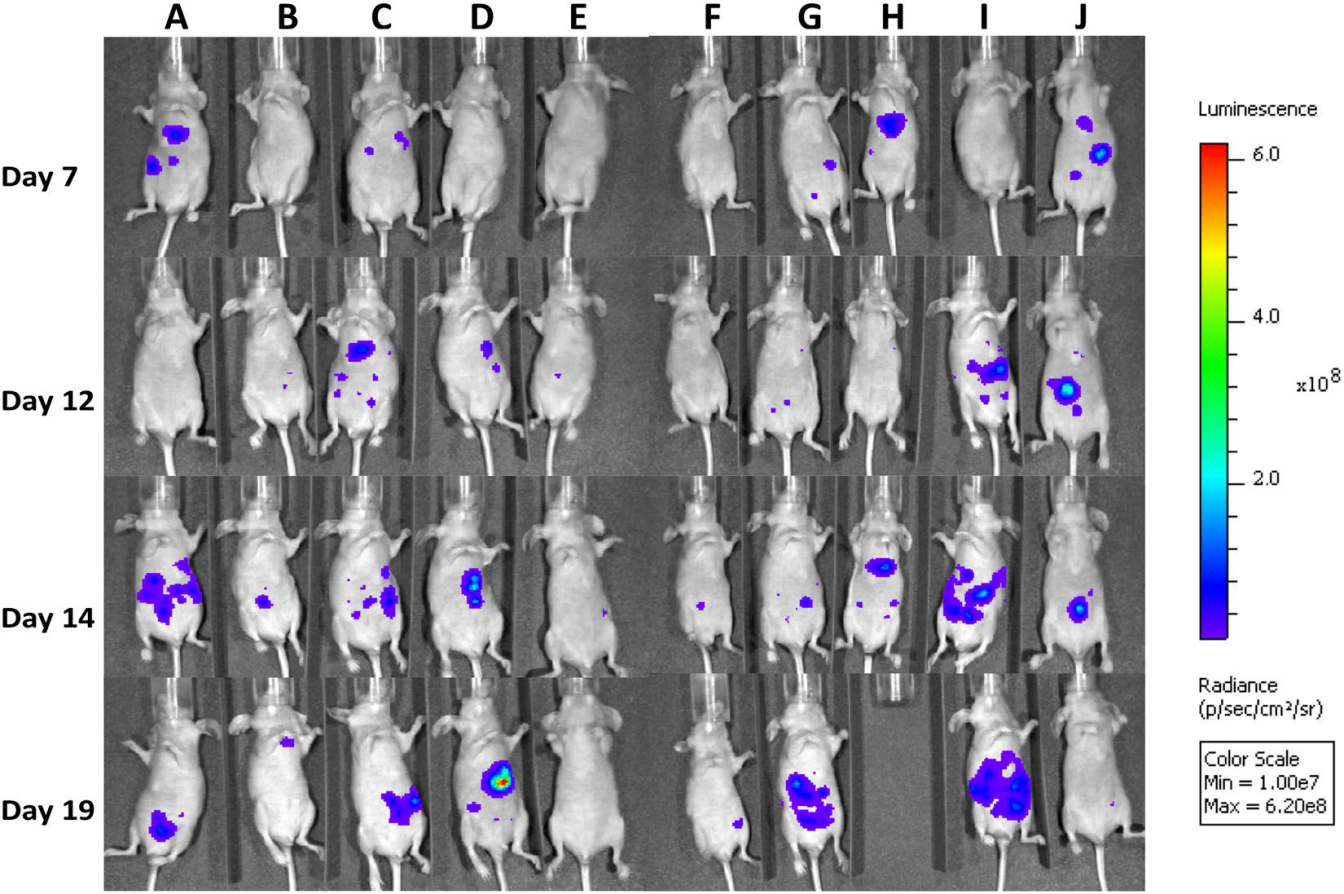
Longitudinal bioluminescence imaging on days 7, 12, 14 and 19. The scale bar indicated standardised radiance across the imaging sessions. The animal shown in Column E did not show significant bioluminescence signal during the longitudinal imaging sessions and no demonstrable tumour by HF-US or post-sacrifice necropsy. Columns B, F and J showed comparatively low signals and corresponding smaller tumours. All other animals had easily demonstrable liver tumours. Animal H was sacrificed on day 16 due to the display of abnormal behaviour.

In addition to HF-US imaging, bioluminescence imaging (BLI) was also performed up to twice weekly (Fig. 5), demonstrating an additional imaging modality for longitudinal monitoring of tumour burden in this model.

## Discussion

To the best of our knowledge, this is the first known demonstration of non-surgical, orthotopic models of human CRCLM and primary CCA using ultrasound-guided intrahepatic injection. This method was minimally invasive thereby reducing risk from surgical complications, whilst fast and easy to perform resulting in measurable tumour volumes 1–3 weeks later. No loss in body mass or changes to normal behaviour were noted at any point during the experiments, with the exception of one CCA mouse at day-16. The use of a 29-gauge needle to inject cells resulted in no visible wound, an advantage over surgical inoculation which would require stitches, resulting in less inflammation and minimal healing time with this method. It has been widely suggested that pain and resulting stress can impact experimental outcomes resulting in poor reliability and reproducibility of experiments involving animals^24^. This model is therefore, an excellent example of the National Centre for the Replacement, Refinement and Reduction of Animals in Research principles involving refinement of technique and development of techniques which minimise the ‘pain, suffering, distress or lasting harm’, directly improving welfare of animals in research^25^. This model represents a refinement of current technique with fewer animals required than with traditional orthotopic models of CRC, as tumour take rates were high and subsequent growth could be monitored longitudinally and non-invasively.

Tumour burden consisted of obvious, large tumours at/near the injection site, with minimal dissemination into the abdominal cavity, a distinct advantage for interventional studies. The necessity of Matrigel may be cell line dependent, it was not necessary for tumour growth and was not used for the CCA cell line TFKLuc2B2, but was however shown to improve SW620 orthotopic rates from 40% to 73–100%. Matrigel is comprised of extracellular matrix proteins, collagen IV and growth factors which can be used to improve cell engraftment in difficult to grow cell lines or primary patient-derived cancer cells^26,27^. Due to the small volumes that could be injected into the intrahepatic space, reduced cell numbers could be used (compared to subcutaneous tumour models) and the Matrigel may have provided a more favourable environment for growth. The Matrigel may also have held the cells in place by the formation of a plug (Matrigel solidifies rapidly above 10°C) therefore preventing leakage of cells from the injection site, as seen in the group without Matrigel. Recently, a group has successfully demonstrated surgical intrahepatic inoculation of hepatocellular carcinoma cells with 100% take rates, however, 50% of these also showed abdominal and pelvic tumours from leakage during intrahepatic injection or potentially a cell line specific result^28^. The non-surgical transplantation of CRC cells into liver has not been done before, but orthotopic pancreatic and bladder cancer models have been successfully developed using ultrasound-guided injection, demonstrating the diversity of models which can be created using minimally invasive methods^29,30^.

HF-US and BLI were used to identify the location of tumours and to monitor the progress of tumour growth *in vivo*. Both are relatively cheap, quick and safe imaging modalities as demonstrated here. Without the use of imaging, the outcome may not be known until end-point dissection and histological analysis, which would be time-consuming and require many more mice to determine appropriate end-points. As shown here, end-point liver mass can also be used to quantitate the degree of CRCLM tumour growth, as livers with tumours increased in mass significantly compared to those without. Other imaging modalities such as BLI (as shown here), magnetic resonance imaging, micro computed tomography or fluorescence^31–33^ may also be used for *in vivo* tumour volume assessment but may not be as economical, rapid or as accurate as HF-US for measurement of tumour volumes *in vivo*^34^.

Orthotopic tumours grow differently to the commonly used subcutaneous models and therefore the importance of using a model which takes into consideration the cancers natural microenvironment is vital for faster and more reliable clinical translation. The formation of large single tumours could reflect late-stage CRC, where large tumours in the liver would require de-bulking (i.e. obstructing portal vein) via adjuvant chemotherapy or radiation, prior to surgical resection. This model provides a promising experimental tool for future testing of anti-cancer agents for the management of metastatic CRC or primary CCA with the possibility of primary liver tumours such as hepatocellular carcinoma, hepatoblastoma or liver metastases from other cancers. The use of syngeneic cell lines in immunocompetent mice or patient-derived cells in immunodeficient mice should also be possible using this method and requires further evaluation.

In summary, this method has successfully demonstrated the development of a minimally-invasive model of CRCLM and CCA. The relatively quick and reliable formation of tumours allows for ease of monitoring of tumour volume via HF-US or BLI, and therefore a means of testing efficacy of novel therapeutics quickly and easily. This model provides a promising experimental tool for future testing of anti-cancer agents in an orthotopic setting.

## Methods

### Cell culture

The SW620 cell line was originally obtained from the American Type Culture Collection (ATCC). Human extrahepatic CCA cells TFK-1 (originally derived from mid-bile duct tumour^35^) were transfected with luciferase for bioluminescence imaging, clones were selected and one was termed TFKLuc2B2. Cells were regularly screened for mycoplasma and were short random repeat (STR) DNA profile authenticated. Cells were maintained in vented T150 tissue culture flasks (Corning Life Sciences, UK) using Roswell Park Memorial Institute medium 1640 (RPMI Medium 1640, Gibco™) supplemented with 10% (v/v) foetal calf serum (FCS) (Invitrogen, UK) at 37°C in a 5% CO_2_ atmosphere. Cells were passaged at 70–80% confluence by washing with sterile phosphate buffered saline (PBS) pH 7.4 (Invitrogen, UK) before being harvested by trypsinisation. Culture medium was added back into the flask to block the action of trypsin before cells were collected and centrifuged at 400 g for 5 minutes using an Eppendorf™ 5810 R centrifuge (Eppendorf™ Hamburg, Germany). Cells were re-suspended in PBS and the total cell count was obtained manually using a Neubauer haemocytometer, and the concentration adjusted using PBS. SW620 cells were further adjusted to 5 × 10^5^ cells/ml using ice-cold Matrigel (Geltrex™ LDEV-free reduced growth factor basement membrane matrix without phenol red, Life Technologies) or PBS immediately prior to injection. TFKLuc2B2 cells were adjusted to 1 × 10^8^ cells/ml using PBS prior to injection.

### Animals

BALB/c nude mice were originally obtained from Charles River Laboratories (Kent, UK) and maintained in-house. All mice were maintained under high health status conditions and were specific pathogen free status. Animals were housed in individually ventilated cages with access to food and water *ad libitum*. All procedures were approved by the UK Home Office and carried out according to the Animals (Scientific Procedures) Act 1986.

### HF-US imaging

HF-US was performed weekly in the CRCLM model and at 3 weeks for the CCA model to measure tumour volume. Using a Vevo 770 HF-US system (Fujifilm VisualSonics, Inc, Ontario, Canada) a 40 MHz (RM-704) transducer (VisualSonics, Inc, Ontario, Canada) was held above the animal in supine position^36^, and imaged in B mode. Vevo 770 version 3 software (Fujifilm VisualSonics, Inc, Ontario, Canada) was used for post-acquisition 3D reconstructions and used to calculate tumour volumes^37^. A video showing HF-US imaging of a CRCLM tumour is included in the Online Resource 4.

### Bioluminescence imaging (BLI)

For *in vivo* BLI of CCA tumours, 150 mg/kg of D-Luciferin (Promega, Madison, WI, USA) was administered intraperitoneally five minutes prior to imaging using an IVIS Spectrum (PerkinElmer, Inc. Waltham, MA, USA). Auto-exposure settings were used with a maximum exposure time of one minute. Living image software version 4.4 (PerkinElmer, Inc. Waltham, MA, USA) was used to analyse bioluminescence signals. Standardised regions of interest were used to calculate average radiance (photons/second/cm^2^/steradian) across imaging sessions.

### Histology

Immediately after sacrifice by cervical dislocation, livers were collected and washed in ice-cold PBS, weighed and fixed by immersion in 4% (w/v) paraformaldehyde. Tissues were blocked in paraffin wax, 5 µm sections cut and stained with Haematoxylin and Eosin (H&E, Sigma, UK) for visual examination. Images were obtained using an Eclipse E1000 light microscope and NIS-Elements BR software (Nikon Instruments Inc., Tokyo, Japan).

### Immunohistochemistry

CD31-positive blood vessels were stained following the manufacturer’s instructions. In brief, sections were deparaffinised and heat-mediated antigen retrieval in 10 mM citrate buffer (pH6) was performed for 10 minutes. Endogenous peroxidases were blocked with 0.3% v/v hydrogen peroxide followed by blocking of endogenous avidin/biotin (Vectorlabs, Burlingame, USA). Sections were then incubated for 1-hour with the primary antibody (1:20 rat anti-mouse CD31, clone SZ31, Dianova GmbH, Germany). After washing, a biotinylated anti-rat secondary antibody was applied for 30 minutes (1:200 Dako, UK). ABC/HRP solutions (ABC/HRP Complex Kit, Vectorlabs, Burlingame, USA) were then applied and visualised with 3,3′-Diaminobenzidine (DAB, Dako, UK). Slides were scanned using an Aperio slide scanner (Leica Biosystems, Wetzlar, Germany) at × 20 magnification. Ten 0.25 mm^2^ boxes were placed randomly throughout the tumour section using RandomSpot software version 6.02^38^ and the number of CD31 positive vessel were counted manually. Microvessel density was calculated as the number of CD31 positive vessels per mm^2^.

### Statistical analyses

All statistical analyses were performed using GraphPad Prism 7 (^©^2017 GraphPad Software, Inc., CA, USA). The type of analysis used in each experiment and statistical significance are shown in figure legends.

## Supplementary information

Supplementary Information

Online Resource 1

Online Resource 2

Online Resource 4

## References

[1] UK bowel cancer key facts. *Cancer Research UK*, http://www.cancerresearchuk.org/cancer-help/type/bowel-cancer. (Accessed: 12th May 2018)

[2] Riihimaki M, Hemminki A, Sundquist J, Hemminki K (2016). Patterns of metastasis in colon and rectal cancer. Sci. Rep..

[3] Public Health England’s National Cancer Intelligence Network (NCIN) Age-standardised incidence rates, one- and five-year survival, all patients diagnosed with upper gastrointestinal cancers, England. https://www.cancerresearchuk.org/about-cancer/bile-duct-cancer/survival. (Accessed: 24th October 2018) (2015).

[4] Chambers A, Groom A, MacDonald I (2002). Dissemination and growth of cancer cells in metastatic sites. Nat. Rev. Cancer.

[5] Sheth KR, Clary BM (2005). Management of hepatic metastases from colorectal cancer. Clinics Colon Rectal Surg..

[6] Oh BY, Hong HK, Lee WY, Cho YB (2017). Animal models of colorectal cancer with liver metastasis. Cancer Lett..

[7] Mcintyre RE, Buczacki SJA, Arends MJ, Adams DJ (2015). Mouse models of colorectal cancer as preclinical models. BioEssays.

[8] Manzotti C, Audisio RA, Pratesi G (1993). Importance of orthotopic implantation for human tumors as model systems: Relevance to metastasis and invasion. Clin. Exp. Metastasis.

[9] Flatmark K, Mælandsmo GM, Martinsen M, Rasmussen H, Fodstad Ø (2004). Twelve colorectal cancer cell lines exhibit highly variable growth and metastatic capacities in an orthotopic model in nude mice. Eur. J. Cancer.

[10] Céspedes MV (2007). Orthotopic microinjection of human colon cancer cells in nude mice induces tumor foci in all clinically relevant metastatic sites. Am. J. Pathol..

[11] Thalheimer A (2009). The intraportal injection model: A preclinical animal model for hepatic metastases and tumor dissemination in human colon cancer. Eur. Surg. Res..

[12] Saxena M, Christofori G (2013). Rebuilding cancer metastasis in the mouse. Mol. Oncol..

[13] Heijstek MW (2005). Mouse Models of Colorectal Cancer and Liver Metastases. Dig. Surg..

[14] Farazi PA (2006). Chronic bile duct injury associated with fibrotic matrix microenvironment provokes cholangiocarcinoma in p53-deficient mice. Cancer Res..

[15] Praet MM, Roels HJ (1984). Histogenesis of cholangiomas and cholangiocarcinomas in thioacetamide fed rats. Exp. Pathol..

[16] Sirica AE (2008). A novel ‘patient-like’ model of cholangiocarcinoma progression based on bile duct inoculation of tumorigenic rat cholangiocyte cell lines. Hepatology.

[17] Dai L, Lu C, Yu XI, Dai L-J, Zhou JX (2015). Construction of orthotopic xenograft mouse models for human pancreatic cancer. Exp. Ther. Med..

[18] Zhao X, Li L, Starr T, Subramanian S (2017). Tumor location impacts immune response in mouse models of colon cancer. Oncotarget.

[19] Hackl C (2013). Metronomic oral topotecan prolongs survival and reduces liver metastasis in improved preclinical orthotopic and adjuvant therapy colon cancer models. Gut.

[20] Wang M (2017). Role of tumor microenvironment in tumorigenesis. J. Cancer.

[21] Sharpless NE, DePinho RA (2006). The mighty mouse: Genetically engineered mouse models in cancer drug development. Nat. Rev. Drug Discov..

[22] Hewitt RE (2000). Validation of a model of colon cancer progression. J. Pathol..

[23] Bruguera M, Aranguibel F, Ros E, Rodes J (1978). Incidence and clinical significance of sinusoidal dilatation in liver biopsies. Gastroenterology.

[24] Balcombe JP, Barnard ND, Sandusky C (2004). Laboratory routines cause animal stress. Contemp. Top. Lab. Anim. Sci..

[25] Prescott MJ, Lidster K (2017). Improving quality of science through better animal welfare: The NC3Rs strategy. Lab Anim. (NY)..

[26] Gock Michael, Kühn Florian, Mullins Christina Susanne, Krohn Mathias, Prall Friedrich, Klar Ernst, Linnebacher Michael (2016). Tumor Take Rate Optimization for Colorectal Carcinoma Patient-Derived Xenograft Models. BioMed Research International.

[27] Pretlow TG, Delmoro CM, Dilley GG, Spadafora CG, Pretlow TP (1991). Transplantation of human prostatic carcinoma into nude mice in matrigel. Cancer Res..

[28] Rao Quan, You Abin, Guo Zhenglong, Zuo Bingfeng, Gao Xianjun, Zhang Ti, Du Zhi, Wu Chenxuan, Yin HaiFang (2016). Intrahepatic Tissue Implantation Represents a Favorable Approach for Establishing Orthotopic Transplantation Hepatocellular Carcinoma Mouse Models. PLOS ONE.

[29] Huynh AS (2011). Development of an orthotopic human pancreatic cancer xenograft model using ultrasound guided injection of cells. PLoS One.

[30] Jäger W (2013). Ultrasound-Guided Intramural Inoculation of Orthotopic Bladder Cancer Xenografts: A Novel High-Precision Approach. PLoS One.

[31] Park Jeong Youp, Murakami Takashi, Lee Jin Young, Zhang Yong, Hoffman Robert M., Bouvet Michael (2016). Fluorescent-Antibody Targeting of Insulin-Like Growth Factor-1 Receptor Visualizes Metastatic Human Colon Cancer in Orthotopic Mouse Models. PLOS ONE.

[32] Magistri, P. *et al*. *In vivo* bioluminescence-based monitoring of liver metastases from colorectal cancer: An experimental model. *J. Microsc. Ultrastruct*. (in press) (2017). 10.4103/JMAU.JMAU_51_18 PMC6753694 31548925

[33] Lee WY, Hong HK, Ham SK, Kim CI, Cho YB (2014). Comparison of colorectal cancer in differentially established liver metastasis models. Anticancer Res..

[34] Ramasawmy Rajiv, Johnson S. Peter, Roberts Thomas A., Stuckey Daniel J., David Anna L., Pedley R. Barbara, Lythgoe Mark F., Siow Bernard, Walker-Samuel Simon (2016). Monitoring the Growth of an Orthotopic Tumour Xenograft Model: Multi-Modal Imaging Assessment with Benchtop MRI (1T), High-Field MRI (9.4T), Ultrasound and Bioluminescence. PLOS ONE.

[35] Saijyo S (1995). Establishment of a new extrahepatic bile duct carcinoma cell line, TFK-1. Tohoku J. Exp. Med..

[36] Abdelrahman MA (2012). High-frequency ultrasound for *in vivo* measurement of colon wall thickness in mice. Ultrasound Med. Biol..

[37] Ingram, N. *et al*. The use of high-frequency ultrasound imaging and biofluorescence for *in vivo* evaluation of gene therapy vectors. *BMC Med. Imaging* **13** (2013). 10.1186/1471-2342-13-35 PMC3831818 24219244

[38] Wright AI, Grabsch HI, Treanor DE (2015). RandomSpot: A web-based tool for systematic random sampling of virtual slides. J. Pathol. Inform..

