# Supplementary material for: Development of orthotopic tumour models using ultrasound-guided intrahepatic injection
Source: Sci Rep. 2019 Jul 9;9:9904. doi: 10.1038/s41598-019-46410-6 (PMC6616610; doi:10.1038/s41598-019-46410-6)
Supplement: Supplementary file 1 — Supplementary Information [file 41598_2019_46410_MOESM1_ESM.pdf]

Online Supplementary Material

Development of orthotopic tumour models using ultrasound-guided intrahepatic injection

L E McVeigh[1*], I Wijetunga[1], N Ingram[1], G Marston[1], R Prasad[2], A F Markham[1], P L Coletta[1]

[1]Leeds Institute of Medical Research, St James's University Hospital, Leeds, LS9 7TF UK
[2]Department of Hepatobiliary and Transplant Surgery, St. James's University Hospital, Leeds, LS9 7TF UK *Corresponding author drlauramcveigh@gmail.com

Online Resource 1 (Video)
HF-US guided intrahepatic injection of human CRC cells. A BALB/c nude mouse was injected with a 40µl bolus of 20,000 SW620 human CRC cells in PBS:Matrigel (1:1 v/v) using HF-US. A needle tip can be seen penetrating the skin and liver, cells injected, and the needle removed. Taken in B-mode using a 40 MHz transducer. Video in real-time.

Online Resource 2 (Video)
HF-US guided intrahepatic injection of human CCA cells. A BALB/c nude mouse was injected with a 50µl bolus of 5 million TFKLuc2B2 cells in PBS using HF-US. The gallbladder (centre screen) was used as a landmark for HF-US guided percutaneous injection into the liver. A needle tip can be seen penetrating the skin and liver, cells injected, and the needle removed. Taken in B-mode using a 40 MHz transducer. Video in real-time.

Online Resource 3 (Image)
BALB/c nude mouse with human CCA orthotopic tumour, 19-days post inoculation. Out of the 9 mice with orthotopic tumours, one animal (shown) also had a discrete intra-peritoneal tumour (PT) and subcutaneous tumour (ST) at the injection site (tumours circled with dashed line).

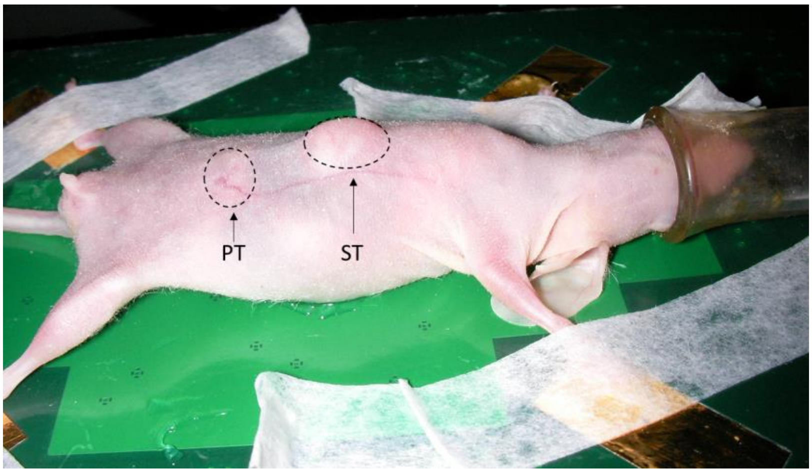

Online Resource 4 (Video)
HFUS video of an intrahepatic human CRC tumour (SW620), 20-days post inoculation. Taken in B-mode using a 40 MHz transducer. Video in real-time.
